# TiGER: A database for tissue-specific gene expression and regulation

**DOI:** 10.1186/1471-2105-9-271

**Published:** 2008-06-09

**Authors:** Xiong Liu, Xueping Yu, Donald J Zack, Heng Zhu, Jiang Qian

**Affiliations:** 1 Wilmer Institute, Johns Hopkins University School of Medicine, Maumenee Building 844, 600 N. Wolfe Street, Baltimore, MD 21287, USA; 2 Department of Molecular Biology and Genetics, Johns Hopkins University School of Medicine, Baltimore, MD 21287, USA; 3 Department of Neuroscience, Johns Hopkins University School of Medicine, Baltimore, MD 21287, USA; 4 McKusick-Nathans Institute of Genetic Medicine, Johns Hopkins University School of Medicine, Baltimore, MD 21287, USA; 5 Department of Pharmacology, Johns Hopkins University School of Medicine, 733 N. Broadway, Baltimore, MD21205, USA

## Abstract

**Background:**

Understanding how genes are expressed and regulated in different tissues is a fundamental and challenging question. However, most of currently available biological databases do not focus on tissue-specific gene regulation.

**Results:**

The recent development of computational methods for tissue-specific combinational gene regulation, based on transcription factor binding sites, enables us to perform a large-scale analysis of tissue-specific gene regulation in human tissues. The results are stored in a web database called TiGER (Tissue-specific Gene Expression and Regulation). The database contains three types of data including tissue-specific gene expression profiles, combinatorial gene regulations, and cis-regulatory module (CRM) detections. At present the database contains expression profiles for 19,526 UniGene genes, combinatorial regulations for 7,341 transcription factor pairs and 6,232 putative CRMs for 2,130 RefSeq genes.

**Conclusion:**

We have developed and made publicly available a database, TiGER, which summarizes and provides large scale data sets for tissue-specific gene expression and regulation in a variety of human tissues. This resource is available at [1].

## Background

A detailed understanding of how genes are expressed and regulated in different tissues can help elucidate the molecular mechanisms of tissue development and function. The approximately 25,000 genes in the human genome demonstrate dramatic diversity in terms of expression levels, both temporally and spatially. Despite this diversity, the expression of all genes is controlled by a relatively small number (<2,000) of transcription factors (TFs). These TFs usually work in specific combination to regulate individual genes [2,3]. A number of databases have been created to facilitate studies of gene expression and regulation. For example, dbEST [4] is a database of expressed sequence tags (ESTs) from a number of organisms; GNF SymAtlas [5] and BodyMap [6] are databases that store human and mouse tissue gene expression profiles; TRANSFAC, TRANSCOMPEL and TRED [7,8] are databases that store information about transcriptional regulation. Some databases, such as CGED [9] and PEDB [10], allow users to access gene expression information derived from either human cancer tissues or one particular tissue (e.g., prostate). However, for a researcher who is interested in tissue-specific gene regulation and would like to examine possible cis-regulatory elements for a gene, a database dedicated to comprehensive information about tissue-specific gene regulation is desirable.

To address this need, we have developed a new database called TiGER (Tissue-specific Gene Expression and Regulation) based on our previous analyses of tissue-specific genes, TFs and cis-regulatory modules (CRMs) for 30 human tissues [3,11]. TiGER should not be confused with the earlier TIGR databases [12] on regulation in microbes, plants and humans. TiGER provides simple search engines so as to permit the users to visualize or download information through a standard web browser. More specifically, the TiGER database has the following features:

• A large set of data on both tissue-specific genes and tissue-specific transcriptional regulatory elements: The database contains tissue-specific expression profiles for ~20,000 UniGene genes, combinatorial regulation for 7,341 interacting TF pairs, and 6,232 cis-regulatory modules for tissue-specific genes.

• Flexible search capability: The database provides three views (gene view, TF view, and tissue view) to allow users to conveniently retrieve information about genes, TFs or tissues of interest. For example, users can simply type a gene ID (e.g., RefSeq) to retrieve the EST profile and CRM detections. Users can also select a tissue name to retrieve a list of genes preferentially expressed in the tissue.

• Convenient accessibility: The database provides visualizations of the gene expression profiles, TF interactions and CRM detections. Sortable summary tables, links to raw data and links to external databases are also provided for user reference.

The rest of the paper will describe the database content and illustrate the utility of the database in tissue-specific gene regulation.

## Construction and Content

TiGER contains three types of data including tissue-specific gene expression profiles, TF interactions and CRMs. The data are organized as a relational database with a user-friendly interface. The following is a detailed description of the database content.

### Tissue-specific gene expression profiles

**Figure 1:**
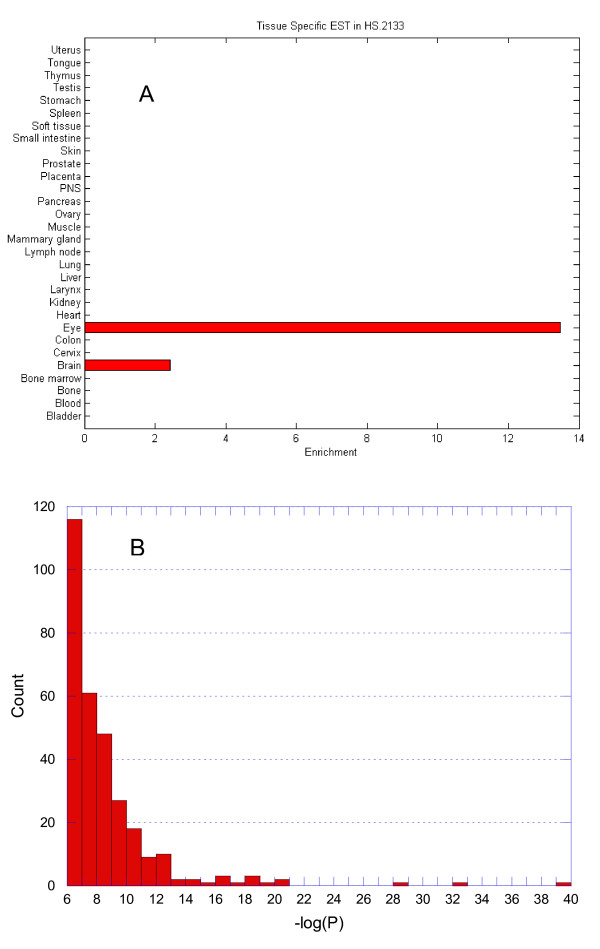
**Tissue-specific gene expression and TF interactions.** (A) An example of gene expression profile. The gene RPE65 (RefSeq ID: NM_000329; UniGene ID: Hs.2133; Ensembl ID: ENSG00000116745) is preferentially expressed in the eye with an expression enrichment value greater than 13. (B) Distribution of -log_10_(p) values for 307 tissue-specific TF interactions in the eye. The most significant is the interaction between FOXJ2 and POU3F2, with a -log_10_(p) value greater than 39 (p-value < 10^-39^).

The ~5.3 millions human EST sequences map to ~54,000 UniGene clusters [4,13]. Previously in [3] we calculated the gene expression pattern for each UniGene in 30 human tissues based on NCBI EST database. We identified 7261 tissue-specific genes for the 30 tissues based on the expression enrichment (EE) and statistical significance. On average, based on these definitions, each tissue expresses ~290 tissue-specific genes [3]. Figure 1A shows the expression profile for the eye-specific gene RPE65 (RefSeq ID: NM_000329; UniGene ID: Hs.2133; Ensembl ID: ENSG00000116745).

### Tissue specific TF interactions

We have developed a method to identify interacting TFs based on patterns of co-occurrence of pairs of DNA binding sites [3]. This method predicts two TFs interact with each other if their binding sites have over-represented co-occurrence in the promoters of tissue-specific genes and the distances (in unite of base pair) between two sites are significantly different from random expectation (as indicated by a small p-value). Using this method, we predicted 9060 tissue-specific TF interactions, around 300 for each tissue. The predicted interactions include many known TF interactions (e.g., MYOD and MEF2 are known to regulate muscle-specific genes) as well as novel interactions. To evaluate these results, we use known interactions as positive control due to the scarcity of tissue-specific interaction. More than 40% of the known interactions are recovered, with 84-fold enrichment compared to the expected. Figure 1B shows the distribution of -log_10_(p) values for the 307 eye-specific TF interactions. The most significant is the interaction between FOXJ2 and POU3F2, with a p-value less than 10^-39^. These two TFs together regulate many eye-specific genes, including RPE65.

### Detection of CRMs

CRMs are the central cis-elements that control gene expression [14]. Previously we developed a method to predict CRMs based on TF interactions [11]. This method calculates the interaction strength between two TF binding sites and then derives an empirical "potential energy" for each TF binding site. Using this method, we generated energy profiles for the promoter sequences of tissue-specific genes. An energy level less than -1 indicates the existence of a TF module. We have sensitivity of 12% and enrichment of 10 using known regulatory regions as positive control.

**Figure 2:**
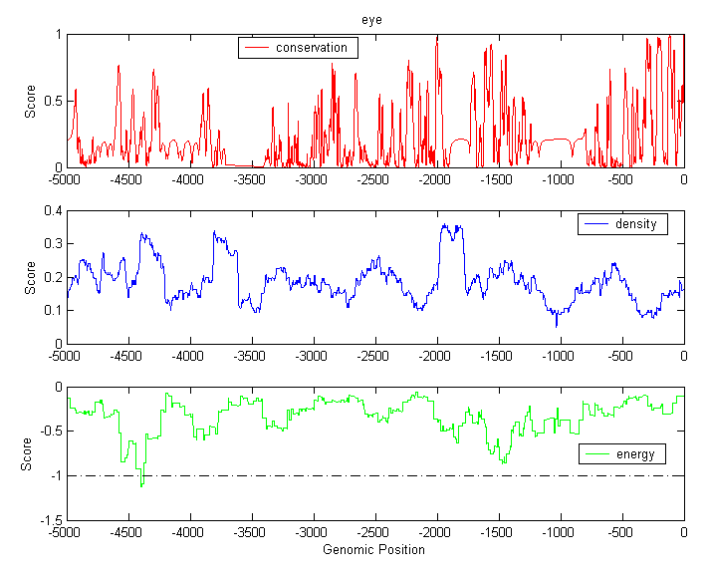
**Conservation profile, density profile and energy profile for the eye-specific gene BFSP1 (RefSeq ID: NM_001195; UniGene ID: Hs.129702; Ensembl ID: ENSG00000125864).** The energies less than -1 indicate the existence of TF modules. The upper panel depicts the conservation scores of the regions between 5 k upstream to translational start site. The middle panel shows the density of all known TF binding sites in a sliding window along the region. The bottom panel shows the potential energy based TF interactions. The dashed line is the thresholds for determining a cis-regulatory module.

Figure 2 illustrates an example of the predicted CRMs for the eye-specific gene BFSP1 (RefSeq ID: NM_001195; UniGene ID: Hs.129702; Ensembl ID: ENSG00000125864). We show the evolutionary conservation, TF binding site density, and potential energy in the plot. The density was calculated by counting the number of all known TFBS in a sliding window (200 bp). The conservation score was obtained from UCSC genome database [15,16]. The transcription start site (TSS) is based on RefSeq. By comparing the conservation and energy profile, we can see that the predicted cis-regulatory modules are not always located in conserved regions. Also, the discrepancy in the density and energy profiles implies the importance of identifying the sets of relevant TFs.

## Utility and Discussion

TiGER is constructed for free access and use. The downloadable data formats include standard .txt text files and .png images. The data contents are configured into three views (saved queries): gene view, TF view, and tissue view, to allow users to conveniently retrieve information relevant to genes, TFs or tissues of interest.

### Gene view

There are three major database entities in the gene view: (1) "EST" entity that stores enrichment values in 30 tissues for each gene; (2) "CRM" entity that stores the conservation profile, the density profile, and the energy profile used for CRM detections in the promoter region of each gene; and (3) "GeneCode" entity that stores the mapping between UniGene, RefSeq and gene symbol.

The gene view allows users to retrieve information through a simple search engine by entering a UniGene gene, a RefSeq gene or a gene symbol. The query results include a gene description, a plot of the EST profile, a list of tissues in which the gene is preferentially expressed, a plot of the three profiles used in CRM detection, and download links to the EST and CRM profiles. Links to external databases such as NCBI, UCSC Genome Browser, and GeneCard, are also included for user references.

### TF view

There is one major database entity called "TF-Partner" in the TF view. This entity stores all factors that interact with a given TF, the tissue in which the interaction occurs and the significance (-log_10_(p)) of the interaction.

The TF view allows users to retrieve TF interactions by entering a TF name. The query results include a summary table of TF interactions, a link to the raw interaction data, and a pie chart which illustrates the distribution of tissues in which the TF interactions occur.

### Tissue view

The tissue view contains three database entities: (1) "TSS-Genes" entity that stores genes preferentially expressed in each of the 30 tissues; (2) "TSS-TFs" entity that stores interactions between TFs in each of the 30 tissues; and (3) "TSS-CRMs" entity that stores CRM modules in the promoter regions of tissue-specific genes. More specifically, the "TSS-Genes" entity contains four attributes including RefSeq gene ID, gene symbol, enrichment values, and descriptions. The "TSS-TFs" entity contains three attributions including the two participating TFs and the significance of interaction. The "TSS-CRMs" entity contains eight attributes including the chromosome ID, RefSeq gene ID, CRM start and end positions, transcription start position and orientation, minimum energy, and a list of TFs that regulate the gene.

**Figure 3:**
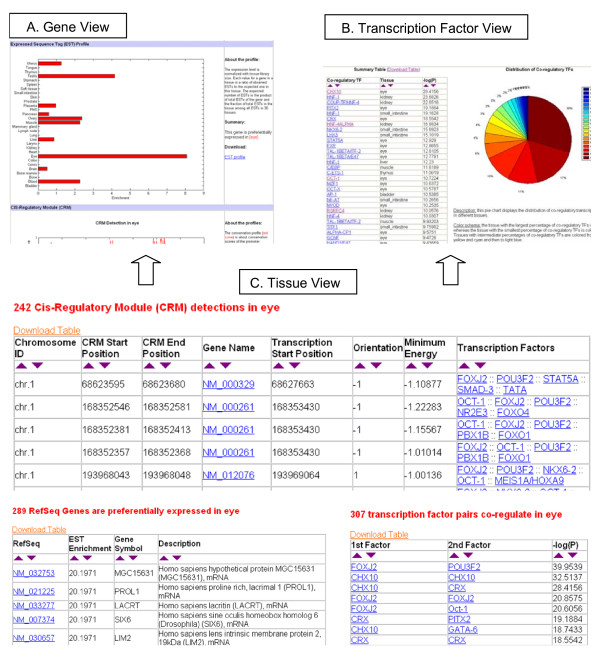
**A diagram of the TiGER views, illustrating three ways a user may search for tissue-specific gene expression and combinatorial regulation.** In (A), the user could type a gene name and obtain a view of gene expressions and crm profiles. In (B), the user could type a TF name and obtain a view of co-regulations. In (C), the user could select a tissue name and obtain a view of gene expressions, crm detections and TF interactions.

To retrieve information for a specific tissue, users can simply select a tissue from a drop-down menu provided in the tissue view. The query results include a summary table of genes specific to the tissue, a summary table of TF interactions and a summary table of CRM modules. These tables are instances of "TSS-Genes", "TSS-TFs", and "TSS-CRMs," respectively. Links to the gene view and the TF view are embedded in the summary tables to provide an integrated environment of query and visualization (see Figure 3).

The query interfaces (views) are implemented as Java servlets which dynamically query the underlying database entities. TiGER operates under an Apache web server and an Apache Tomcat engine on a SuSe Linux system. The plots of gene expression profiles, TF interactions and CRM detections are pre-generated in Matlab.

## Conclusion

We performed a large-scale analysis of gene expression, TF interaction and CRM detection in 30 human tissues. The results are stored in a web-enabled database called TiGER and configured so as to permit users to visualize or download the results through a standard web browser.

There are fundamental issues relating to the computational prediction of human gene regulation. Future research will include both prediction models on gene regulation and analysis tools for interpreting prediction results. As more experimental data accumulates related to the nature of TF-DNA interactions, we plan to further develop our predictions on tissue-specific TF interactions. We also plan to extend our work on CRM detection by relating regulatory elements with temporal (e.g., development) and spatial (e.g., cell types) attributes. As new predictions on tissue-specific gene regulation accumulate, the TiGER database will need to be further expanded and modified. We will update the content of the database on a regular basis. We also plan to develop tools relating TiGER data to other available gene expression and regulation data for integrative analysis.

## Availability and requirements

Project name: TiGER

Project home page: [1]

Operating system(s): Platform independent.

Programming language: none.

License: no restriction.

Any restrictions to use by non-academics: no restriction.

## Authors' contributions

XL, XY and JQ conceived the construction of the database. XL developed the database interface. XY generated the data. JQ supervised the development and implementation. DJZ and HZ helped to interpret the results. XL and JQ drafted the paper, and all authors read and approved the final manuscript.
